# Prognostic characteristics in hormone receptor-positive advanced breast cancer and characterization of abemaciclib efficacy

**DOI:** 10.1038/s41523-018-0094-2

**Published:** 2018-12-18

**Authors:** Angelo Di Leo, Joyce O’Shaughnessy, George W. Sledge, Miguel Martin, Yong Lin, Martin Frenzel, Molly C. Hardebeck, Ian C. Smith, Antonio Llombart-Cussac, Matthew P. Goetz, Stephen Johnston

**Affiliations:** 1 Hospital of Prato, Istituto Toscano Tumori, Prato, Italy; 2 Baylor University Medical Center, Texas Oncology, US Oncology, Dallas, TX USA; 3 Stanford University, Stanford, CA USA; 4 Instituto De Investigacion Sanitaria Gregorio Marañon, Ciberonc, Geicam, Universidad Complutense, Madrid, Spain; 5 Eli Lilly and Company, Indianapolis, IN USA; 6 FISABIO—Hospital Arnau Vilanova, Valencia, Spain; 7 Mayo Clinic, Rochester, MN USA; 8 The Royal Marsden NHS Foundation Trust, London, UK

## Abstract

CDK4 & 6 inhibitors have enhanced the effectiveness of endocrine therapy (ET) in patients with advanced breast cancer (ABC). This paper presents exploratory analyses examining patient and disease characteristics that may inform in whom and when abemaciclib should be initiated. MONARCH 2 and 3 enrolled women with HR+, HER2- ABC. In MONARCH 2, patients whose disease had progressed while receiving ET were administered fulvestrant+abemaciclib/placebo. In MONARCH 3, patients received a nonsteroidal aromatase inhibitor+abemaciclib/placebo as initial therapy for advanced disease. A combined analysis of the two studies was performed to determine significant prognostic factors. Efficacy results (PFS and ORR in patients with measurable disease) were examined for patient subgroups corresponding to each significant prognostic factor. Analysis of clinical factors confirmed the following to have prognostic value: bone-only disease, liver metastases, tumor grade, progesterone receptor status, performance status, treatment-free interval (TFI) from the end of adjuvant ET, and time from diagnosis to recurrence. Prognosis was poorer in patients with liver metastases, progesterone receptor-negative tumors, high grade tumors, or short TFI (<36 months). Benefit (PFS hazard ratio, ORR increase) from abemaciclib was observed in all patient subgroups. Patients with indicators of poor prognosis had the largest benefit from the addition of abemaciclib. However, in MONARCH 3, for patients with certain good prognostic factors (TFI ≥ 36 months, bone-only disease) ET achieved a median PFS of >20 months. These analyses identified prognostic factors and demonstrated that patients with poor prognostic factors derived the largest benefit from the addition of abemaciclib.

## Biomarkers: Prognostic factors indicate likely beneficiaries of cell cycle–blocking therapy

A series of clinical factors may help identify those patients with hormone receptor-positive breast cancer who are most likely to benefit from a newly approved cell cycle–blocking drug. Angelo Di Leo from the Hospital of Prato, Italy, and colleagues retrospectively analyzed data from two phase III trials that evaluated the CDK4 & 6 inhibitor abemaciclib against a placebo administered in combination with either fulvestrant endocrine therapy or an aromatase inhibitor. Pooling the results, the researchers found that patients with certain indicators of poor prognosis—including liver metastases, progesterone receptor negativity, high-grade tumors, or short treatment-free intervals following the completion of adjuvant endocrine therapy—benefited most from abemaciclib, as measured by progression-free survival. The findings, if confirmed in prospective trials, could help inform personalized drug choices for patients.

## Introduction

Over 70% of metastatic breast cancers are hormone receptor-positive (HR+) and are treated with sequential endocrine-based therapies.^1–4^ Endocrine therapies (ETs) may initially be efficacious and well-tolerated in a substantial proportion of patients with HR+ breast cancer. However, for the majority, ET will eventually become ineffective.^2^

Efforts to improve the effectiveness of ET by adding medicines that target potential mechanisms of resistance are ongoing.^5–12^ One of the most successful approaches is the combination of cyclin-dependent kinase 4 & 6 (CDK4 & 6) inhibitors with ET.^3,4,7–10,12^ These combinations have improved progression-free survival (PFS) and objective response rates (ORR) in patients with HR+ advanced breast cancer (ABC), both as initial therapy and after progression on ET. Since none of the Phase III studies reported thus far permitted crossover between treatment arms upon progressive disease, the relative value of upfront CDK4 & 6 therapy versus therapy on progression is unknown.^7–10,12^ Furthermore, no predictive markers for HR+ breast cancer have been identified for this class of medicines.^13,14^ Prior studies have described potential prognostic factors for patients with HR+ ABC, including metastatic site (visceral, liver, bone-only) and prior sensitivity to ET (disease-free interval/treatment-free interval [TFI]).^6,12,15–18^ In addition, tumor-specific prognostic factors in the adjuvant setting include progesterone receptor (PgR) expression and tumor grade.^19^ However, the implications of these factors in guiding treatment decisions for the use of ET alone versus in combination with CDK4 & 6 inhibitors need further exploration.

Given the complexity of these treatments, the identification of patient and tumor characteristics that can help inform when to use CDK4 & 6 inhibitors in the treatment paradigm and in which patients is a subject of considerable interest.^13,20,21^ CDK4 & 6 inhibitor trials published to date have demonstrated treatment benefit for the addition of a CDK4 & 6 inhibitor to ET across all patient subgroups.^7-10,12,22^ The present analyses of abemaciclib aim to determine independently prognostic subgroups, characterize the benefit of the addition of abemaciclib to endocrine therapy in these subgroups, and then determine those which derived the largest benefit from abemaciclib and those for which endocrine monotherapy may be an appropriate initial treatment. This approach may inform tailoring of treatment choices to individual patients.

**Fig. 1:**
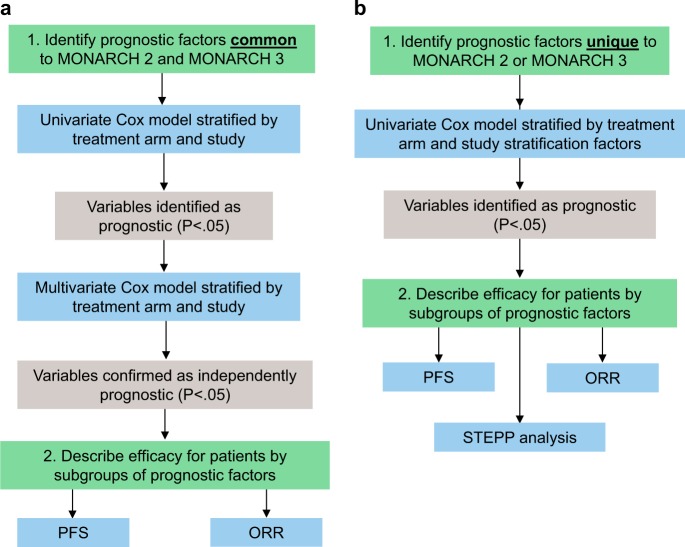
Method for identification of prognostic factors. Identification of prognostic factors that are common for MONARCH 2 and MONARCH 3 **a** and that are unique for MONARCH 2 or MONARCH 3 **b**. PFS progression-free survival, ORR objective response rate, STEPP subpopulation treatment effect pattern plot

These analyses use data from two Phase III studies in patients with HR+, HER2− ABC in which abemaciclib plus ET significantly improved outcomes for patients as initial therapy (MONARCH 3) and in disease that progressed while receiving ET (MONARCH 2).^10,12^ A two-step approach was employed that first identified independent prognostic characteristics in the MONARCH 2 and 3 studies (Fig. 1). Where possible, data were pooled across studies to maximize the power to detect prognostic factors. The second step described the outcomes of patients who received ET alone versus ET plus abemaciclib. Thus, the treatment effect (PFS hazard ratio [HR] and ORR increase) of adding abemaciclib to ET can be interpreted in the context of the performance of endocrine monotherapy in the same population.

## Results

### Demographic, clinical, and histological factors

Patients in MONARCH 2 were enrolled from August 7, 2014 to December 29, 2015 and in MONARCH 3 from November 18, 2014 to November 11, 2015. This subgroup analysis uses data from the final PFS analyses of MONARCH 2 and MONARCH 3. Both studies are still blinded for overall survival. Patient disposition for MONARCH 2 and MONARCH 3 is described in the CONSORT diagram (Supplementary Fig. 1).^10,12^

Overall, baseline characteristics were generally balanced between treatment arms (Supplementary Table 1). Of the factors examined, race, Eastern Cooperative Oncology Group performance status (ECOG PS), bone-only disease, visceral disease, presence of liver metastases, PgR status, tumor grade, and number of organs at baseline were found to be significant, whereas age, presence of lung metastases, presence of pleural metastases, and prior neoadjuvant or adjuvant chemotherapy were not (Table 1).

**Table 1:** Prognostic analysis of demographic, disease, and histological factors

Subgroup	Univariate *p*-value	Multivariate *p*-value^b^
*MONARCH 2 and MONARCH 3* ^*a*^		
Age	0.9343	N/A
Race	0.0046	N/A
ECOG PS	0.0069	0.0020
Bone-only disease	<.0001	0.0007
Visceral disease	<.0001	N/A
Liver metastases	<.0001	<.0001
Lung metastases	0.5067	N/A
Pleural metastases	0.4619	N/A
PgR status	0.0205	0.0070
Grade	0.0016	0.0017
No. of organs at baseline	0.0003	N/A
Prior neoadjuvant or adjuvant chemotherapy	0.3204	N/A
*MONARCH 2 only* ^c^		
Number of lines of ET	0.2944	N/A
Last Line of ET	0.6673	N/A
ET Resistance	0.1142	N/A
*MONARCH 3 only* ^c^		
Treatment-free interval	0.0225	N/A
Time from diagnosis to recurrence	0.0224	N/A
De novo metastatic disease	0.9266	N/A

*ECOG PS* Eastern Cooperative Oncology Group performance status, *ET* endocrine therapy, *N/A* not applicable, *PgR* progesterone receptor

^a^Pooled univariate and multivariate analyses were stratified by study and treatment arm

^b^Patients with any missing potential baseline prognostic factors were removed from the multivariate analysis. The stepwise selection used *p*-value < 0.05 as the criterion for adding a variable and *p*-value ≥ 0.05 for dropping a variable

^c^MONARCH 2 only and MONARCH 3 only were stratified by study stratification factors and treatment arm

Some significant prognostic factors may potentially be related and redundant predictors of prognosis. Thus, a multivariate analysis was performed using a stepwise Cox proportional hazards model. All factors previously identified as significant, with the exception of race, visceral disease, and number of involved organs at baseline, remained significantly associated with prognosis (Table 1), indicating that these variables are necessary and the significance of one factor is independent of the others.

**Fig. 2:**
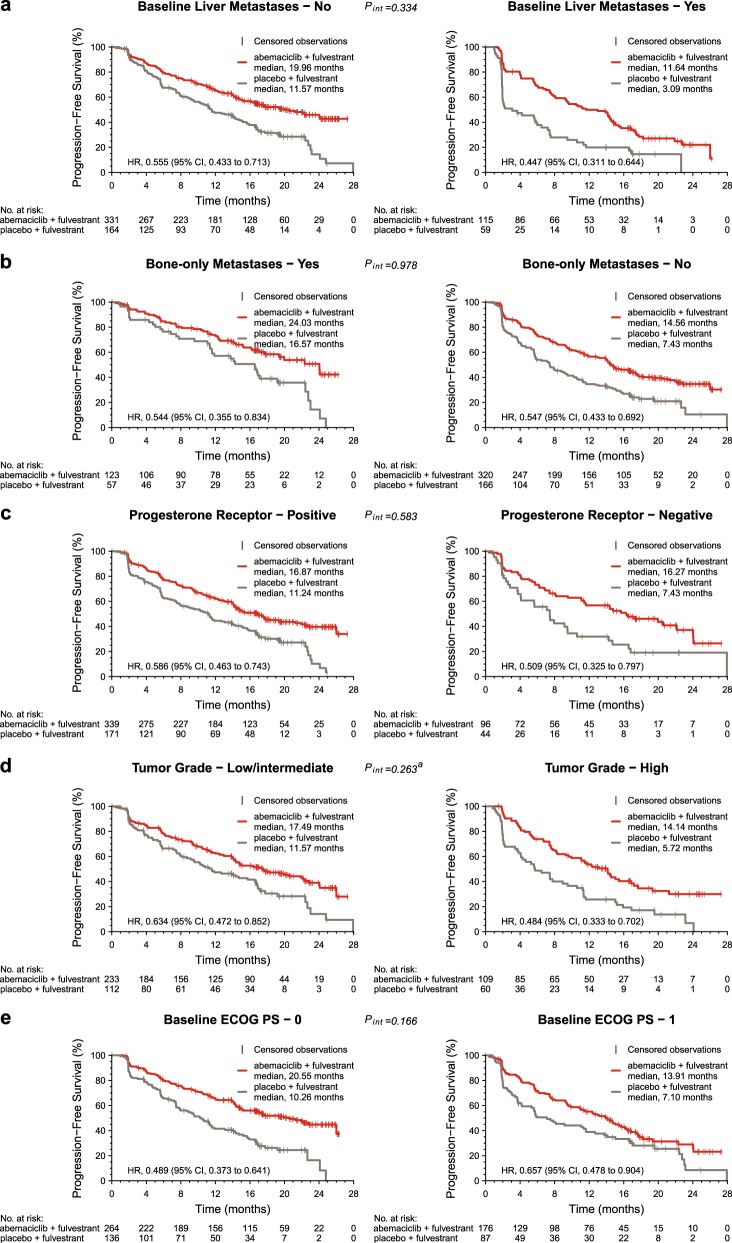
Kaplan–Meier plots of progression-free survival in patient subgroups in MONARCH 2. Subgroups include patients without and with liver metastases **a**, with or without bone-only metastases **b**, progesterone receptor-positive or progesterone receptor-negative status **c**, low/intermediate or high tumor grade **d**, and baseline Eastern Cooperative Oncology Group performance status (ECOG PS) of 0 or 1 **e**. CI confidence interval, HR hazard ratio. ^a^Interaction of tumor grade has been adjusted for removal of patients with unknown tumor grade

**Fig. 3:**
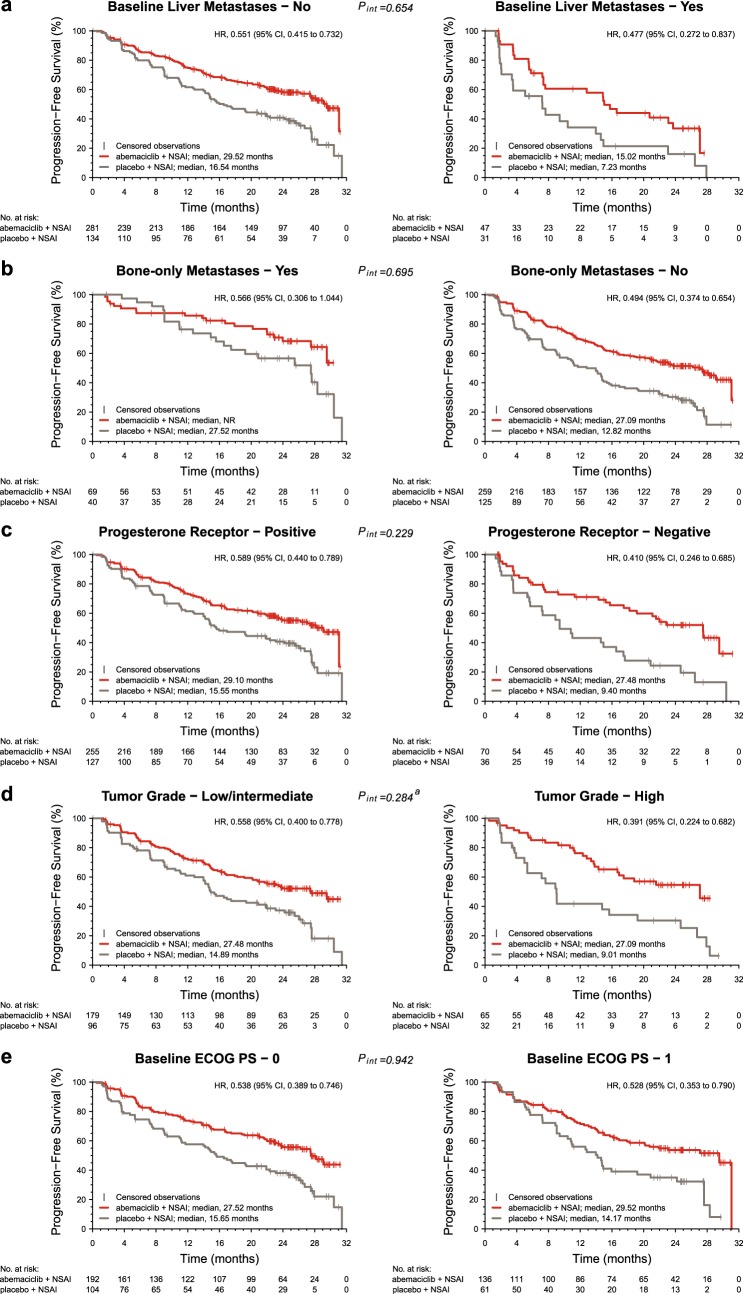
Kaplan–Meier plots of progression-free survival in patient subgroups in MONARCH 3. Subgroups include patients without and with liver metastases **a**, with or without bone-only metastases **b**, progesterone receptor-positive or progesterone receptor-negative status **c**, low/intermediate or high tumor grade **d**, and baseline Eastern Cooperative Oncology Group performance status (ECOG PS) of 0 or 1 **e**. CI confidence interval, HR hazard ratio, NR not reached, NSAI nonsteroidal aromatase inhibitor. ^a^Interaction of tumor grade has been adjusted for removal of patients with unknown tumor grade

Analysis within each significant subgroup revealed differences in the PFS of the control arms (Figs. 2 and 3). Patients with liver metastases had the shortest PFS in the setting of endocrine monotherapy, with a median PFS of 7.2 months in MONARCH 3 and 3.1 months in MONARCH 2. Shorter PFS was also observed in the control arm in patients with either PgR-negative or high grade tumors. In contrast, the median PFS for patients treated with endocrine monotherapy was substantially longer for patients with an ECOG PS of 0 (MONARCH 3: 15.7 months; MONARCH 2: 10.3 months) or bone-only disease (MONARCH 3: 27.5 months; MONARCH 2: 16.6 months).

When evaluating PFS, adding abemaciclib to ET provided consistent benefit across patient subgroups defined by the five identified significant prognostic factors (Figs. 2 and 3). No significant interactions between treatment and these five factors were identified.The addition of abemaciclib provided the largest benefit (HRs in the range of 0.4–0.5 and ORR increases typically over 30%) in patients with liver metastases, PgR-negative tumors, or high grade tumors, across the two studies. Conversely, in patients with bone-only disease treated in the context of the MONARCH 3 trial, despite a HR of 0.566, the Kaplan–Meier curves overlapped during the first 9–12 months of treatment.

### Prior endocrine treatment

In MONARCH 3, patients were ET naïve or had a disease-free interval >12 months from the completion of adjuvant ET (median duration of adjuvant ET: 60.1 months). To identify prognostic factors related to endocrine sensitivity, a univariate analysis was performed to examine the significance of TFI (<36 or ≥36 months), time from diagnosis to recurrence (TTR) (≤10 or >10 years), and de novo metastatic disease (yes or no). The first two analyses excluded patients with de novo metastatic disease, and TFI included only patients who had received adjuvant ET. Univariate analysis indicated TFI and TTR as significant prognostic factors (Table 1).

In contrast to MONARCH 3, patients in MONARCH 2 had disease that progressed while receiving ET. The factors examined for prognostic significance included number of lines of ET (1 or 2), last line of ET (neoadjuvant/adjuvant or metastatic), and type of ET resistance (primary or secondary).^3,4,10^ However, none were significant in the univariate analysis (Table 1).

**Fig. 4:**
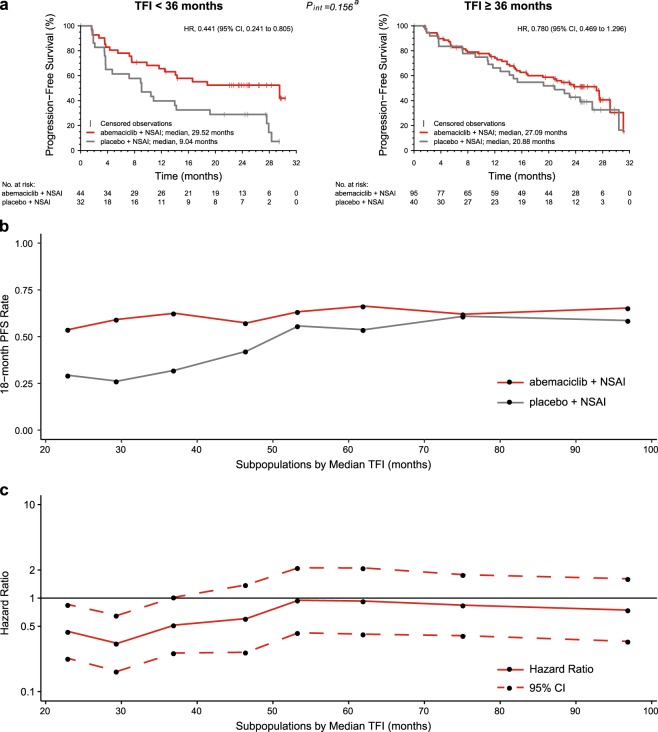
Treatment-free interval (TFI) in MONARCH 3. Kaplan–Meier plots for treatment-free interval < 36 months and ≥36 months **a**. Subpopulation treatment effect pattern plot analysis of treatment-free interval using 18-month progression-free survival rate **b** and hazard ratio (HR) **c**. CI confidence interval, NSAI nonsteroidal aromatase inhibitor. ^a^Interaction for TFI has been adjusted for removal of patients with de novo disease

The MONARCH 3 analysis of TFI dichotomized at a 36-month cut-off indicated that patients with a longer TFI had a longer PFS (Fig. 4a). Subgroup analysis based on TFI indicated that patients with a TFI < 36 months derived a large benefit from adding abemaciclib to NSAI, in addition to an increase in ORR of 32.11% (Supplementary Table 2).

To further examine the prognostic value of TFI and the association of the effect of abemaciclib and TFI, independently of the 36-month cut-off, sliding window subpopulation treatment effect pattern plot (STEPP) analyses of the 18-month PFS rate for both arms and the PFS HR were performed. These variables were evaluated across eight overlapping groups of ~80 patients, with patients in each group having similar TFIs and adjacent groups sharing 60 common patients. As TFI increased, the 18-month PFS rate increased in both arms (Fig. 4b), supporting the conclusion that TFI was a relevant prognostic variable. In addition, a shorter TFI was associated with a better treatment effect (HR) with abemaciclib compared to a longer TFI (Fig. 4c).

Similar to TFI, the dichotomized analysis of TTR using a 10-year cut-off showed TTR to be significantly prognostic. However, STEPP analyses of the 18-month PFS rate and PFS HR showed no interpretable pattern, indicating that TTR may not be relevant information in the treatment choice (Supplementary Fig. 2).

## Discussion

This subgroup analysis of the MONARCH 2 and 3 trials evaluates and determines the independent prognostic effects of a large number of pathological and clinical characteristics that can inform the prognosis of patients treated with contemporary endocrine-based therapy. This analysis used a data set derived from over 1000 patients who participated in the MONARCH 2 and 3 Phase III studies.

Previously reported subgroup analyses of studies combining CDK4 & 6 inhibitors with ET have concluded that all subgroups benefit from the addition of CDK4 & 6 inhibitors.^7–10,22^ However, clinical decision-making encompasses communicating to patients the absolute benefit of a given therapy. These results suggest that the absolute benefit provided by abemaciclib may be meaningful across a spectrum of pathological and clinical characteristics, thus potentially identifying a group of patients for whom combined treatment with abemaciclib and ET could provide clinically significant gains. Conversely, these data also suggest that these factors may identify a group of patients for whom endocrine monotherapy may be an appropriate initial therapy. Unlike other reports of subgroup analyses in which subgroup variables were arbitrarily selected or selected on preconceived biases, our approach was to assess all available demographic and clinical variables to identify those characteristics that were independently prognostic. By first identifying prognostic subgroups, the relative treatment effect (PFS HR and ORR increase) of adding abemaciclib to ET in a prognostic subgroup may be interpreted in the context of the median PFS and ORR of endocrine monotherapy in that population.

A caveat of this approach is that it was limited to the possible prognostic factors that were identifiable within the MONARCH 2 and 3 databases. A two-step approach was used: first, identifying independent prognostic variables derived from the entire population regardless of treatment assignment, and second, describing the treatment effects of endocrine monotherapy and ET combined with abemaciclib in each of the identified prognostic subgroups.

The first step of the exploratory analysis identified two groups of clinical characteristics of independent prognostic relevance for patients receiving endocrine-based therapy in MONARCH 2 and 3: those characteristics that existed in both populations and those that existed in only one. The former consisted of histological grade (low/intermediate grade conferring better prognosis than high), PgR status (positive conferring a better prognosis than negative), liver metastases (absence conferring a better prognosis than presence), bone-only metastases (presence conferring a better prognosis than absence), and ECOG status (0 conferring a better prognosis than 1). The only prognostic characteristics not common to both studies identified as being independent prognostic variables were TFI and TTR (longer conferring a better prognosis than shorter for both variables) in MONARCH 3. Of note, visceral disease was significantly prognostic in the univariate analysis but not in the multivariate analysis, indicating that presence of liver metastases drove the perceived prognostic value of visceral disease (Table 1 and Supplementary Fig. 3). In addition, primary versus secondary resistance per ESMO guidelines was not found to be significantly prognostic, but it is possible that this test was under powered given only MONARCH 2 patients could be included in this analysis (Table 1 and Supplementary Fig. 4).

The second step of the analysis described the outcomes of patients who received ET alone or ET plus abemaciclib in each of the prognostic subgroups identified within the first step. Abemaciclib conferred substantial benefit in patients regardless of prognosis. However, patients with poor prognostic factors (liver metastases, PgR-negative status, or high grade tumors) consistently derived the largest benefit from the addition of abemaciclib to ET. Furthermore, in MONARCH 3, patients who had received adjuvant ET were evaluated using a STEPP analysis of TFI, demonstrating that those with a shorter TFI derived the largest benefit from the addition of abemaciclib to ET.

The interaction tests between these prognostic factors and treatment effect were not significant. Given the fact that the studies were not designed or powered to detect interactions, this lack of significance should be balanced against the available evidence. Specifically, the differential treatment effect observed in patients with poor prognostic factors (liver metastases, PgR-negative status, or high grade tumors) was consistently better across both MONARCH 2 and 3, lending credibility to the hypothesis that these patients may benefit more from the addition of abemaciclib to ET.

In summary, the results of these exploratory analyses have identified certain key prognostic factors, which can be used to identify groups of patients that derived large and clinically significant benefits from treatment with abemaciclib in both the first and second line settings, regardless of the endocrine partner. Data from patients receiving initial therapy for advanced disease, have led us to hypothesize that there may be a group of patients with clinically indolent disease that can be treated sequentially with endocrine monotherapy (e.g. aromatase inhibitor) followed by the introduction of a CDK4 & 6 inhibitor at progression. It should be noted that in a population with indolent disease detecting a difference in PFS between treatment groups will take an appropriately powered study with much longer follow up. However, groups of patients with more aggressive disease and thus concerning clinical characteristics gained considerable benefit from the addition of abemaciclib to ET. These data are hypothesis generating and will need to be evaluated along with other known pathological and molecular determinants of abemaciclib response in the context of prospective clinical trials. Importantly, these data can provide the groundwork to develop individualized therapy for women with HR+, HER2− breast cancer.

## Patients and methods

### Study design and patients

MONARCH 2 and 3 (NCT02107703 and NCT02246621, respectively) are Phase III, randomized, double-blind trials for women with locally tested HR+, HER2− ABC.^10,12^ MONARCH 2 investigated abemaciclib or placebo plus fulvestrant in patients whose disease had progressed while receiving ET. MONARCH 3 investigated abemaciclib or placebo plus a nonsteroidal aromatase inhibitor (NSAI) as initial therapy.

In both studies, eligible women were aged ≥ 18 years with a 0 or 1 ECOG PS. Patients had measurable disease or non-measurable bone-only disease (blastic, lytic, or mixed) as defined by the Response Evaluation Criteria in Solid Tumors (RECIST) v1.1.^23^ In MONARCH 2, patients had disease that progressed while receiving adjuvant ET, ≤12 months from the end of adjuvant ET, or while receiving ET for ABC. Patients were permitted one prior ET and no prior chemotherapy for ABC. MONARCH 2 allowed any menopausal status (pre-/peri-menopausal women received a gonadotropin-releasing hormone agonist); MONARCH 3 enrolled postmenopausal patients. In MONARCH 3, patients were ET naïve or could have received neoadjuvant or adjuvant ET if the disease-free interval was >12 months from the end of ET. Other inclusion and exclusion criteria were previously described.^10,12^

The MONARCH 2 and MONARCH 3 studies were approved by the local IRBs for the sites participating in the clinical trials. The data in this manuscript are retrospective analyses of the data from these studies. All patients provided written informed consent. The trials were conducted in compliance with the Declaration of Helsinki. Trial protocols were previously disclosed on www.clinicaltrials.gov.

### Randomization and treatment procedures

In MONARCH 2, patients were randomized (2:1) to receive abemaciclib (150 mg twice-daily continuous schedule [or 200 mg prior to protocol amendment]) or matching placebo plus fulvestrant (500 mg per label).^10^ Patients were stratified by metastatic site (visceral, bone-only, or other) and ET resistance (primary or secondary), as defined by European Society for Medical Oncology guidelines.^3,4,10^ In MONARCH 3, patients were randomized (2:1) to receive abemaciclib (150 mg twice-daily continuous schedule) or matching placebo plus NSAI (1 mg anastrozole or 2.5 mg letrozole, daily, per physician’s choice).^12^ Patients were stratified by metastatic site (visceral, bone-only, or other) and prior neoadjuvant or adjuvant ET (AI, no ET, or other).^12^ Dose reductions and interruptions were previously described.^10,12^

### Efficacy measures

Using RECIST v1.1, tumors were evaluated by computed tomography or magnetic resonance imaging at baseline and post-baseline. Scanning frequencies were previously described.^10,12^

### Outcomes

The endpoints of both studies, including investigator-assessed PFS (the primary endpoint of both studies), ORR, and safety results, were previously described.^10,12^

### Statistical analysis

This exploratory post hoc analysis compared the investigator-assessed PFS between abemaciclib plus ET and placebo plus ET among subgroups in the MONARCH 2 and 3 studies. ORRs in patients with measurable disease are provided as supportive data but no formal comparisons between subgroups were made. The sample size, randomization methods, and statistical methods for the primary and secondary endpoints for the original trials were previously described.^10,12^

To identify prognostic variables potentially associated with the performance of endocrine monotherapy or combination therapy, cross-study subgroup analyses were performed for key demographic and clinical variables and those identified in the literature as associated with prognosis: age, race, baseline ECOG PS, bone-only disease, visceral disease, liver metastases, lung metastases, pleural metastases, PgR status (local report), tumor grade (local report), number of involved organs at baseline, and prior neoadjuvant or adjuvant chemotherapy (Fig.1a). Univariate Cox model analysis, stratified by study and treatment arm, was used to evaluate each variable independent of treatment as potentially prognostic. A variable was considered potentially prognostic if the likelihood ratio *p*-value was < 0.05. Subsequently, a multivariate Cox model analysis, was performed including variables found to be potentially prognostic by univariate analysis. Variables were selected in a stepwise fashion, with an entry *p*-value = 0.05 and a retaining *p*-value = 0.05. Only patients with a complete record of included baseline variables were included. For subgroup variables identified as prognostic through multivariate analysis, treatment effects for the addition of abemaciclib to ET were reported for each subgroup within each study. Treatment effects were summarized by study and treatment arm.

Due to differences in entry criteria, and thus study populations, variables associated with sensitivity to ET were evaluated at a study level in a separate analysis from above (Fig. 1b). For MONARCH 2, univariate analysis of number of ETs received (1 versus 2), most recent ET (adjuvant versus metastatic), and level of ET resistance (primary versus secondary) were performed as described above. For MONARCH 3, the variables included TFI from the end of adjuvant ET (<36 versus ≥36 months); TTR (≤10 versus > 10 years); and de novo metastatic disease (yes versus no). No multivariate analysis was performed. For variables found to be prognostic by univariate analysis, treatment effects for the addition of abemaciclib to ET were reported for each subgroup within the relevant study.

To further explore the prognostic value of TFI and TTR, STEPP analyses were performed.^24^ TFI was performed on the subset of patients from MONARCH 3 who previously received adjuvant ET (*n* = 211). TTR was performed on the subset of patients from MONARCH 3 who had recurrent disease (*n* = 292). The analysis was performed using the 18-month PFS rate in each arm as the response. Patients were grouped using *r*_1_ = 60 and *r*_2_ = 80. No inferential statistics were calculated due to difficulties in controlling Type I error with such a sample size using this analysis.^25^ To describe the effect of adding abemaciclib to ET in the same manner, the analysis was repeated using the stratified HR as the response. SAS v9.2 or later (SAS Institute) was used for statistical analyses.

## Electronic supplementary material

Supplemental Material_Di Leo_M2 and 3 Subgroup

## Data Availability

Lilly makes patient-level data available from Lilly-sponsored studies on marketed drugs for approved uses following acceptance for publication. Lilly is one of several companies that provide this access through the website clinicalstudydatarequest.com. Qualified researchers can submit research proposals and request anonymized data to test new hypotheses. Lilly’s data sharing policies are provided on the clinicalstudydatarequest.com site under the Study Sponsors page.

## Supplementary material

**Supplementary information** accompanies the paper on the *npj Breast Cancer* website (10.1038/s41523-018-0094-2).
