# Supplementary material for: Prognostic characteristics in hormone receptor-positive advanced breast cancer and characterization of abemaciclib efficacy
Source: NPJ Breast Cancer. 2018 Dec 18;4:41. doi: 10.1038/s41523-018-0094-2 (PMC6299082; doi:10.1038/s41523-018-0094-2)
Supplement: Supplementary file 1 — Supplemental Material_Di Leo_M2 and 3 Subgroup [file 41523_2018_94_MOESM1_ESM.docx]

**Supplementary Information**

**855** patients assessed for eligibility (MONARCH 2)

**579** patients assessed for eligibility (MONARCH 3)

**142** excluded (MONARCH 2)

**107** did not meet inclusion criteria

**27** refused to participate

**8** for other reasons

**86** excluded (MONARCH 3)

**55** did not meet inclusion criteria

**18** refused to participate

**13** for other reasons

**669** in the ITT population (MONARCH 2)

**493** in the ITT population (MONARCH 3)

**446** analyzed for efficacy (MONARCH 2)

**328** analyzed for efficacy (MONARCH 3)

**441** analyzed for safety (MONARCH 2)

**327** analyzed for safety (MONARCH 3)^a^

**6** lost to follow-up (MONARCH 2)

**1** lost to follow-up (MONARCH 3)

**271** discontinued treatment (MONARCH 2)

**201** discontinued treatment (MONARCH 3)

**446** allocated to abemaciclib + fulvestrant (MONARCH 2)

**441** received allocated treatment

**5** did not receive allocated treatment

**328** allocated to abemaciclib + NSAI (MONARCH 3)

**326** received allocated treatment^a^

**2** did not receive allocated treatment

**223** analyzed for efficacy (MONARCH 2)

**165** analyzed for efficacy (MONARCH 3)

**223** analyzed for safety (MONARCH 2)

**161** analyzed for safety (MONARCH 3)^a^

**223** allocated to placebo + fulvestrant (MONARCH 2)

**223** received allocated treatment

**0** did not receive allocated treatment

**165** allocated to placebo + NSAI (MONARCH 3)

**162** received allocated treatment^a^

**3** did not receive allocated treatment

**Supplementary Figure 1. CONSORT diagram^1,2^**

Abbreviations: ITT, intent-to-treat; NSAI, nonsteroidal aromatase inhibitor

^a^In MONARCH 3, during Cycle 1 a single patient who was randomized to placebo received abemaciclib, and this patient is included in the abemaciclib safety population.

**4** lost to follow-up (MONARCH 2)

**0** lost to follow-up (MONARCH 3)

**178** discontinued treatment (MONARCH 2)

**127** discontinued treatment (MONARCH 3)

**713** patients enrolled (MONARCH 2)

**493** patients enrolled (MONARCH 3)

.


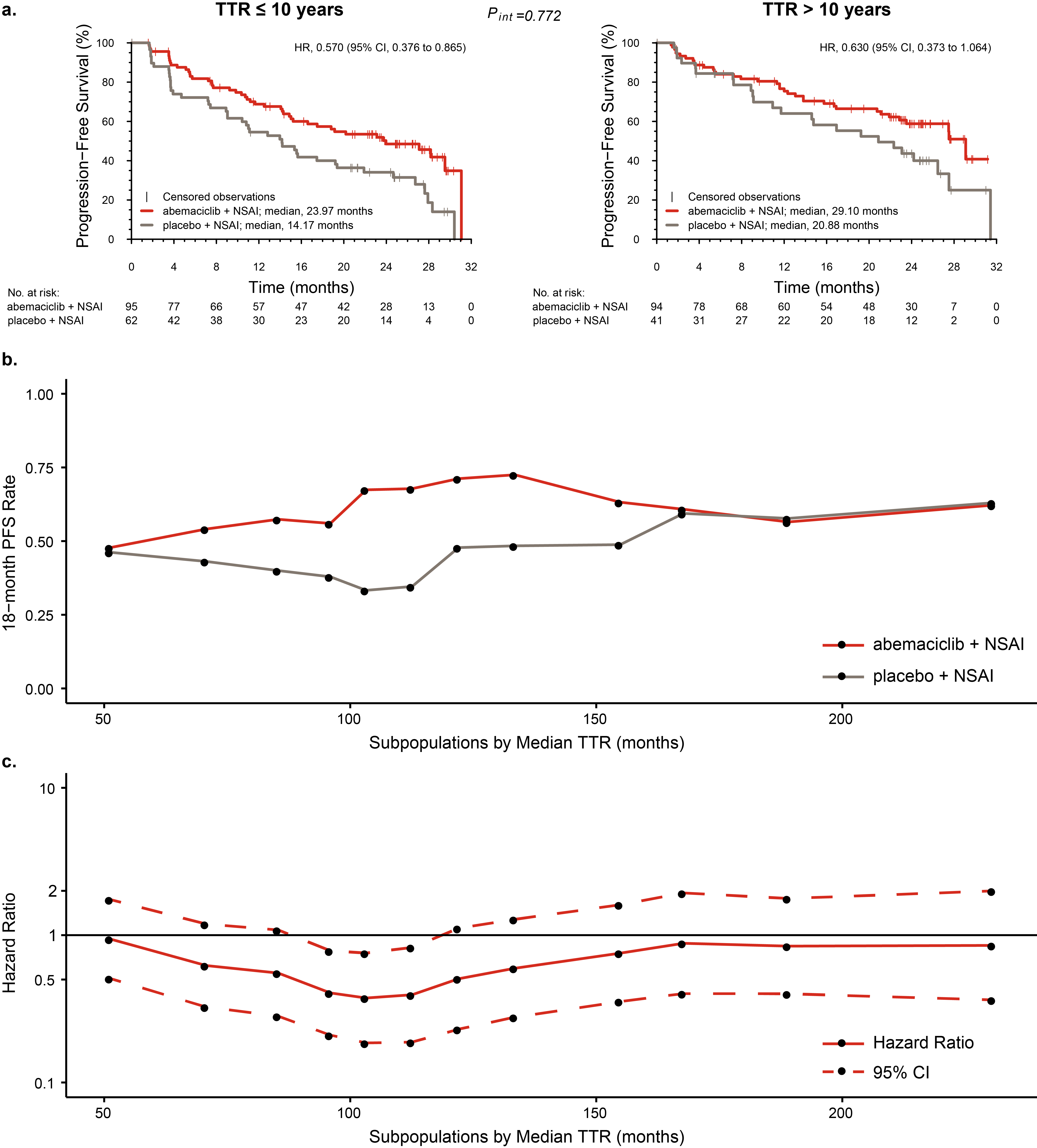


**Supplementary Figure 2. Time from Diagnosis to Recurrence (TTR) in MONARCH 3**Kaplan-Meier plots for TTR ≤10 years and >10 years (a). Subpopulation treatment effect pattern plot analysis of TTR using 18-month progression-free survival rate (b) and hazard ratio (HR) (c). Abbreviations: CI, confidence interval; NSAI, nonsteroidal aromatase inhibitor.

**
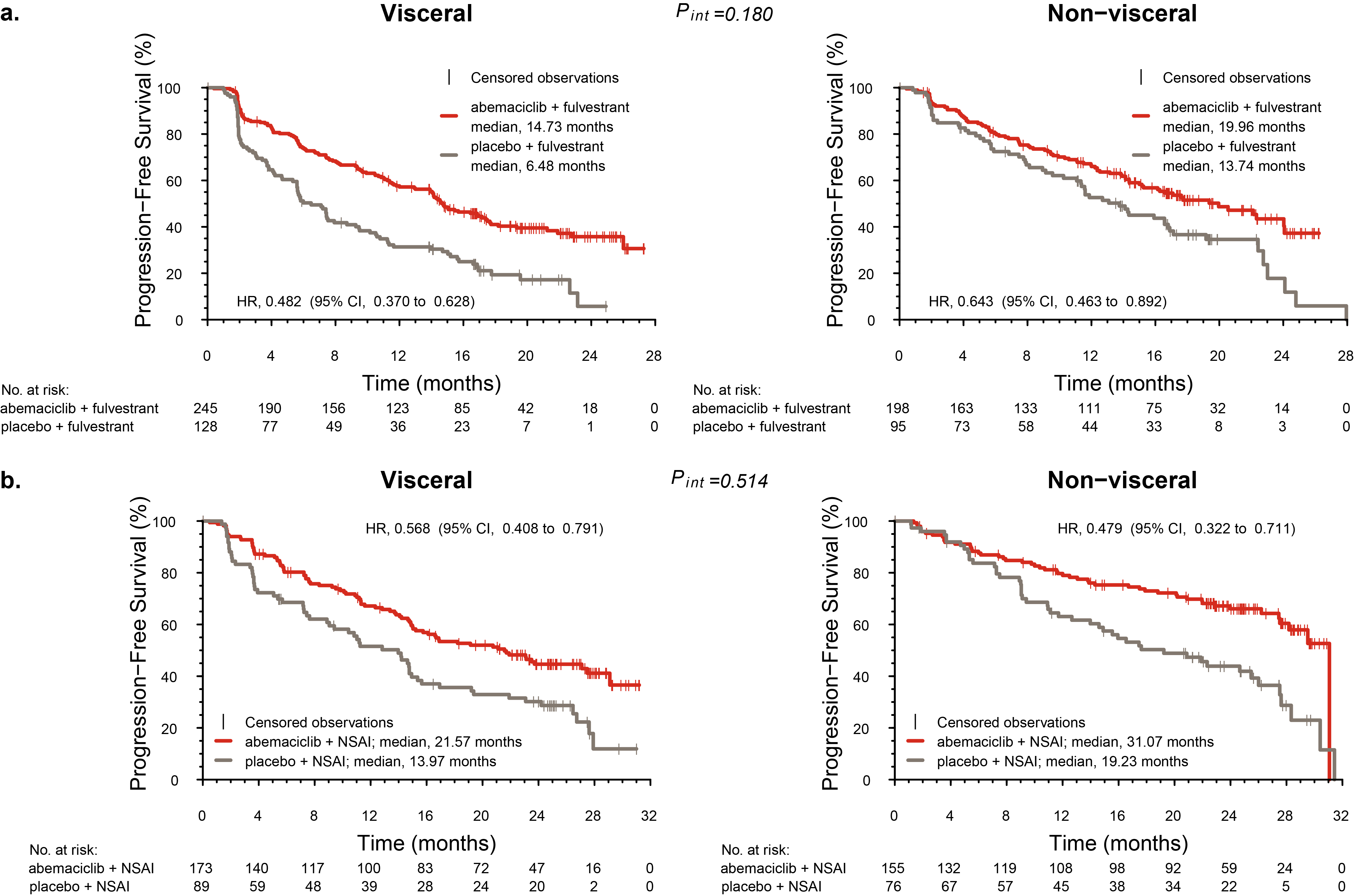
**

**Supplementary Figure 3. Visceral and Non-Visceral Disease**

Kaplan-Meier plots for visceral and non-visceral disease in MONARCH 2 (a) and MONARCH 3 (b). Abbreviations: CI, confidence interval; HR, hazard ratio; NSAI, nonsteroidal aromatase inhibitor.

**
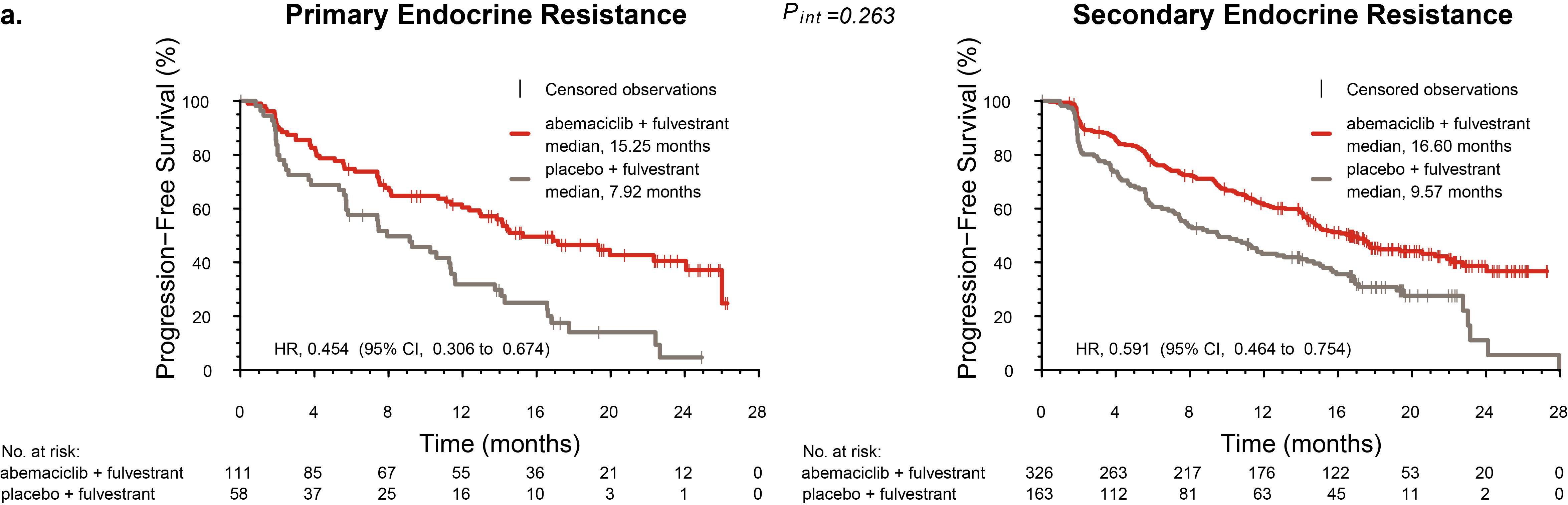
**

**Supplementary Figure 4. Primary and Secondary Endocrine Therapy Resistance in MONARCH 2**Kaplan-Meier plots for primary and secondary resistance per European Society of Medical Oncology guidelines (a).^3,4^ Abbreviations: CI, confidence interval; HR, hazard ratio.

**Supplementary Table 1. Patient and Disease Baseline Characteristics**

| Characteristic | abemaciclib arms | placebo arms |
| --- | --- | --- |
| MONARCH 2 and 3 | (n=774) | (n=388) |
| Median age, years |  |  |
| <65 | 471 (60.9) | 224 (57.7) |
| ≥65 | 303 (39.1) | 164 (42.3) |
| Race^a^ |  |  |
| Caucasian | 423 (54.7) | 238 (61.3) |
| Asian | 252 (32.6) | 110 (28.4) |
| Other | 40 (5.2) | 20 (5.2) |
| ECOG PS^a^ |  |  |
| 0 | 456 (58.9) | 240 (61.9) |
| 1 | 312 (40.3) | 148 (38.1) |
| Bone-only disease^a^ |  |  |
| Yes | 192 (24.8) | 97 (25.0) |
| No | 579 (74.8) | 291 (75.0) |
| Visceral disease^a^ |  |  |
| Yes | 418 (54.0) | 217 (55.9) |
| No | 353 (45.6) | 171 (44.1) |
| Liver metastases |  |  |
| Yes | 162 (20.9) | 90 (23.2) |
| No | 612 (79.1) | 298 (76.8) |
| Lung metastases^a^ |  |  |
| Yes | 245 (31.7) | 121 (31.2) |
| No | 526 (68.0) | 267 (68.8) |
| Pleural metastases^a^ |  |  |
| Yes | 99 (12.8) | 53 (13.7) |
| No | 672 (86.8) | 335 (86.3) |
| PgR status^a^ |  |  |
| Positive | 594 (76.7) | 298 (76.8) |
| Negative | 166 (21.4) | 80 (20.6) |
| Grade |  |  |
| Low/Intermediate | 412 (53.2) | 208 (53.6) |
| High | 174 (22.5) | 92 (23.7) |
| Unknown | 188 (24.3) | 88 (22.7) |
| No. of organs at baseline^a^ |  |  |
| 1 | 280 (36.2) | 127 (32.7) |
| 2 | 211 (27.3) | 109 (28.1) |
| ≥3 | 279 (36.0) | 151 (38.9) |
| Prior neoadjuvant or adjuvant chemotherapy |  |  |
| Yes | 393 (50.8) | 200 (51.5) |
| No | 381 (49.2) | 188 (48.5) |
| MONARCH 2 Only | (n=446) | (n=223) |
| Number of lines of ET^a^ |  |  |
| 1 | 354 (79.4) | 173 (77.6) |
| 2 | 80 (17.9) | 45 (20.2) |
| Last line of ET^a^ |  |  |
| Neoadjuvant or adjuvant | 263 (59.0) | 133 (59.6) |
| Metastatic | 171 (38.3) | 85 (38.1) |
| ET Resistance^b^ |  |  |
| Primary | 111 (24.9) | 58 (26.0) |
| Secondary | 326 (73.1) | 163 (73.1) |
|  |  |  |
| Supplementary Table 1 continued |  |  |
| Characteristic | **abemaciclib arms** | **placebo arms** |
| MONARCH 3 Only | (n=328) | (n=165) |
| Treatment-free interval^c^ |  |  |
| <36 months | 44 (13.4) | 32 (19.4) |
| ≥36 months | 95 (29.0) | 40 (24.2) |
| Time from diagnosis to recurrence^a,d^ |  |  |
| ≤10 years | 95 (29.0) | 62 (37.6) |
| >10 years | 94 (28.7) | 41 (24.8) |
| De novo metastatic disease |  |  |
| Yes | 135 (41.2) | 61 (37.0) |
| No | 193 (58.8) | 104 (63.0) |

Note: Data given as No. (%).

Abbreviations: ECOG PS, Eastern Cooperative Oncology Group performance status; ET, endocrine therapy; PgR, progesterone receptor.

^a^With the exception of grade and treatment-free interval, the percent of patients with missing data for any given characteristic was less than 5%. Analyses of these characteristics excluded patients with missing data. For tumor grade, an unknown category is listed and was included in the analysis due to the large number of patients with missing data for this characteristic.
^b^According to European Society for Medical Oncology Guidelines, primary ET resistance is defined as patients whose disease relapsed while receiving the first 2 years of neoadjuvant or adjuvant ET or progressed while receiving the first 6 months of ET for ABC. Patients without primary ET resistance were defined as having secondary ET resistance.^1,3,4^ Note that 6 patients in the abemaciclib arm and 2 in the placebo arm had not received prior ET.

^c^Treatment-free interval calculated only for patients with prior ET. Treatment-free interval was unknown in 12 patients in the abemaciclib arm and 8 in the placebo arm.

^d^Time from diagnosis to recurrence calculated only for patients who had disease that recurred.

|  | **Control Arm** | | **Abemaciclib Arm** | | **Change in ORR** | |
| --- | --- | --- | --- | --- | --- | --- |
|  | **NSAI**  n/n1 (%) | **fulvestrant**  n/n1 (%) | **NSAI**  n/n1 (%) | **fulvestrant**  n/n1 (%) | **abemaciclib + NSAI** (%) | **abemaciclib  + fulvestrant** (%) |
| **Intent-to-treat** | 60/132 (45.45) | 35/164 (21.34) | 163/267 (61.05) | 153/318 (48.11) | +15.59% | +26.77 |
| **ECOG PS** |  |  |  |  |  |  |
| 1 | 22/50 (44.00) | 14/62 (22.58) | 75/112 (66.96) | 59/120 (49.17) | +22.96 | +26.59 |
| 0 | 38/82 (46.34) | 21/102 (20.59) | 88/155 (56.77) | 94/198 (47.47) | +10.43 | +26.89 |
| **Bone-only disease** |  |  |  |  |  |  |
| No | 54/122 (44.26) | 34/156 (21.79) | 157/254 (61.81) | 148/299 (49.50) | +17.55 | +27.70 |
| Yes | N/A^a^ | N/A^a^ | N/A^a^ | N/A^a^ | N/A^a^ | N/A^a^ |
| **Liver metastases** |  |  |  |  |  |  |
| Yes | 6/30 (20.00) | 9/59 (15.25) | 27/47 (57.45) | 54/111 (48.65) | +37.45 | +33.39 |
| No | 54/102 (52.94) | 26/105 (24.76) | 136/220 (61.82) | 99/207 (47.83) | +8.88 | +23.06 |
| **PgR status** |  |  |  |  |  |  |
| Negative | 8/29 (27.59) | 3/31 (9.68) | 37/61 (60.66) | 29/66 (43.94) | +33.07 | +34.26 |
| Positive | 52/103 (50.49) | 32/126 (25.40) | 125/204 (61.27) | 124/248 (50.00) | +10.78 | +24.60 |
| **Grade** |  |  |  |  |  |  |
| High | 11/28 (39.29) | 10/48 (20.83) | 38/56 (67.86) | 39/76 (51.32) | +28.57 | +30.48 |
| Intermediate/Low | 36/75 (48.00) | 16/82 (19.51) | 91/139 (65.47) | 80/170 (47.06) | +17.47 | +27.55 |
| Unknown | 13/29 (44.83) | 9/34 (26.47) | 34/72 (47.22) | 34/72 (47.22) | +2.39 | +20.75 |
| **TFI** |  |  |  |  |  |  |
| <36 months | 5/22 (22.73) | N/A | 17/31 (54.84) | N/A | +32.11 | N/A |
| ≥36 months | 16/31 (51.61) | N/A | 41/73 (56.16) | N/A | +4.55 | N/A |
| **TTR** |  |  |  |  |  |  |
| ≤10 years | 18/45 (40.00) | N/A | 42/71 (59.15) | N/A | +19.15 | N/A |
| >10 years | 15/33 (45.45) | N/A | 41/69 (59.42) | N/A | +13.97 | N/A |

**Supplementary Table 2. ORR for Patients with Measurable Disease in Patient Subgroups based on Prognostic Factors**

Abbreviations: ECOG PS, Eastern Cooperative Oncology Group performance status; HR, hazard ratio; n, number of responders; n1, total number of patients in each subgroup; N/A, not applicable; NSAI, nonsteroidal aromatase inhibitor; ORR, objective response rate; PgR, progesterone receptor; TFI, treatment-free interval; TTR, time from diagnosis to recurrence.

^a^Response rate not reported for bone-only disease because the majority of lesions were not measurable.

**Supplementary References**

1 Sledge, G. W., Jr. *et al.* MONARCH 2: Abemaciclib in Combination With Fulvestrant in Women With HR+/HER2- Advanced Breast Cancer Who Had Progressed While Receiving Endocrine Therapy. *J Clin Oncol* **35**, 2875-2884, doi:10.1200/jco.2017.73.7585 (2017).

2 Goetz, M. P. *et al.* MONARCH 3: Abemaciclib As Initial Therapy for Advanced Breast Cancer. *J Clin Oncol* **35**, 3638-3646, doi:10.1200/jco.2017.75.6155 (2017).

3 Cardoso, F. *et al.* 3rd ESO-ESMO international consensus guidelines for Advanced Breast Cancer (ABC 3). *Breast* **31**, 244-259, doi:10.1016/j.breast.2016.10.001 (2017).

4 Cardoso, F. *et al.* 3rd ESO-ESMO International Consensus Guidelines for Advanced Breast Cancer (ABC 3). *Ann Oncol* **28**, 16-33, doi:10.1093/annonc/mdw544 (2017).
